# Epicardial adipose tissue, aortic stiffness and myocardial fibrosis in healthy individuals: a quantitative cardiac magnetic resonance study

**DOI:** 10.1186/1532-429X-18-S1-Q4

**Published:** 2016-01-27

**Authors:** Rami Homsi, Daniel K Thomas, Juergen Gieseke, Darius Dabir, Julian A Luetkens, Christian Marx, Daniel Kuetting, Hans H Schild, Alois Martin Sprinkart

**Affiliations:** 1 Radiology, University Hospital, Bonn, Germany; 2 Philips Healthcare, Best, Netherlands

## Background

Epicardial adipose tissue, aortic stiffness and myocardial fibrosis have been linked to cardiovascular risk and disease; however, often different modalities are used for their measurement. Cardiac magnetic resonance (CMR) may be used to determine these parameters in a single examination by the measurement of epicardial fat volumes (EFV), aortic pulse wave velocity (PWV) as a parameter of aortic stiffness, and T1-relaxation time (T1) as a marker for myocardial fibrosis. This quantitative CMR-study was performed to identify relationships between these parameters in healthy individuals and to correlate them with age, body mass index (BMI) and gender.

## Methods

**Figure 1:**
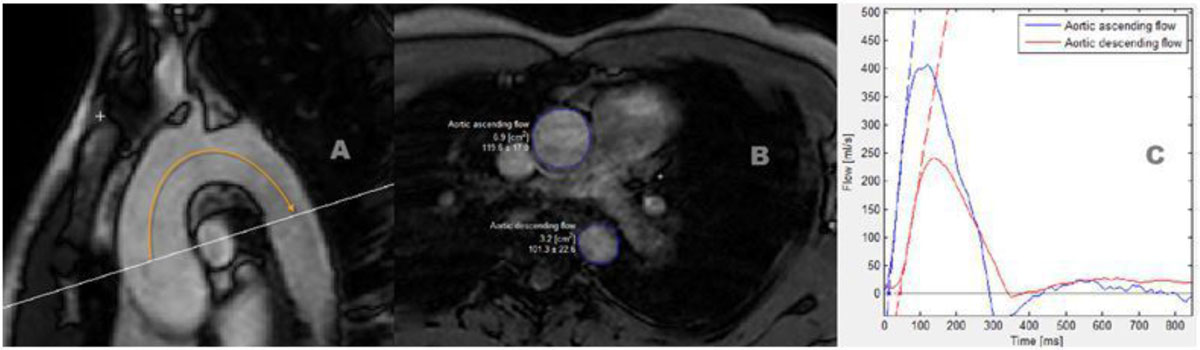
**PWV quantification was performed using a tool implemented in the software Segment (Segment, version 1.9, R3918; {{a href='http://segment.heiberg.se/'}}**http://segment.heiberg.se**{{/a}})**. A: path length of the aortic arch (aortic length = AL) which is the distance between the section through the ascending aorta (AA) and through the proximal descending aorta (AD). B: Region of interest (ROI) in the AA and in the AD in the aortic velocity maps. C: Flow curves along with their respective calculated tangents Transit Time. PWV = AL/TT.

**Figure 2:**
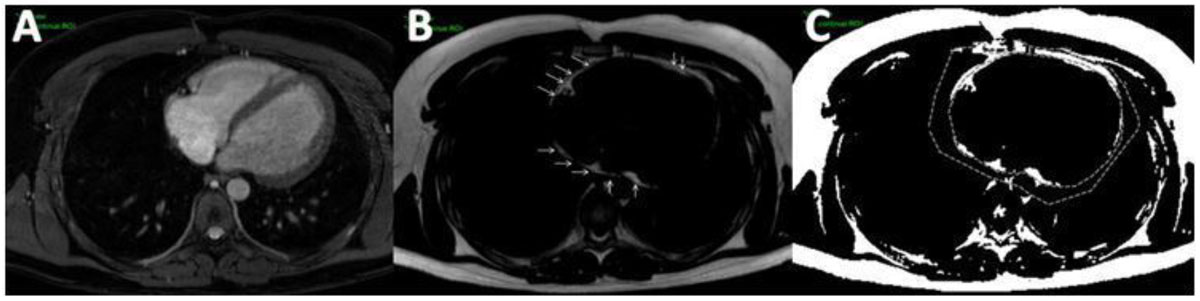
**Dixon image analysis for measurement of epicardial and pericardial fat volume (EFV, PFV in a 21 year old healthy male**. A: Reconstructed water-only image. B: Reconstructed fat-only image with the arrows pointing at the pericardial outline, which corresponding to the region of interest for EFV. C: Segemented fat voxels with transferred regions of interest (ROI) for EFV (inner ROI) and PFV (out ROI).

58 healthy individuals with different age and BMI (29 men and 29 women; mean age 44.7 ± 13.9 years[y]; mean BMI 25.5 ± 4.8 kg/m²) underwent a comprehensive CMR exam (1.5 Tesla MR system, Ingenia, Philips). Native T1-relaxation time for myocardial fibrosis was assessed using the modified Look-Locker Inversion Recovery technique. Aortic PWV as a marker of aortic stiffness was determined 2D-velocity encoded CMR. Quantification of aortic PWV (figure 1) was performed using the software Segment (Segment, version 1.9, R3918; http://segment.heiberg.se). A 3D transversal ECG- and respiratory navigator gated mDixon-sequence was performed for the evaluation of EFV (figure 2). EFV was measured based on fat fraction maps and were normalized to the body surface area.

## Results

There was a correlation between EFV and PWV (mean EFV 44.2 ± 25.0; mean PWV 6.9 ± 1.9 m/s; p < 0.05) independent of age. There were no associations between EFV and T1 and between PWV and T1 (mean T1: 967.5 ± 44.2). Splitting the individuals in two groups (older group > 45 years and younger group < 45 years), the older group (mean age 56.7 ± 8.4) had significantly higher PWV and EFV values (7.9 m/s vs. 6.0 m/s and 54.7 ml/m² vs. 34.5 ml/m²; p < 0.05, each), with no significant difference in T1 or BMI. Splitting the individuals in a group with BMI > 25 kg/m² and BMI < 25 kg/m², the overweighted group (mean BMI 28.7 kg/m²) had significantly higher EFV values (56.1 ± 27.1 vs. 31.5 ± 14.6, p < 0.01) with no significant difference in PWV or T1. Men had higher EFV than women (51.1 ± 35.8 vs. 37.3 ± 21.5) with no differences concerning PWV and T1.

## Conclusions

EFV and aortic PWV were both associated with age. However, EFV and aortic PWV were also associated with each other, independent of age. Proinflammatory markers which may act in epicardial fat and which have been shown to promote atherosclerosis may lead to a higher aortic stiffness in individuals with increased epicardial adipose tissue through similar mechanisms.

Future studies have to concentrate on patients with increased cardiovascular risk and disease in order to evaluate the relationship between epicardial fat volume, aortic stiffness and myocardial fibrosis. CMR provides a valuable tool for such studies and may play an important role in cardiovascular risk stratification and disease.

